# Effects of Trazodone on Testicular Morphology, Sperm Parameters and Contractility of the Distal Cauda Epididymal Duct in Rats

**DOI:** 10.1111/bcpt.70302

**Published:** 2026-09-04

**Authors:** Maria Santana da Silva, Vitória Barros Marques, Fernanda Karoline Oliveira da Silva, Maele Oliveira de Sena, Yanka do Vale Carvalho, Bruna da Silva Figueiredo, Luana Talinne da Costa Gomes, Samia Ellen Alves Fernandes, Edherlayne Bezerra dos Santos Silva, Jackson Nazareno Gomes de Lima, Anna Karynna Alves de Alencar Rocha, Expedito Silva do Nascimento Junior, Elaine Cristina Gavioli, Edilson Dantas da Silva Junior

**Affiliations:** ^1^ Mode of Drug Action Laboratory Federal University of Rio Grande do Norte Natal Rio Grande do Norte Brazil; ^2^ Maurício de Nassau University Center Natal Rio Grande do Norte Brazil; ^3^ Department of Morphology Federal University of Rio Grande do Norte Natal Rio Grande do Norte Brazil; ^4^ Department of Biophysics and Pharmacology Federal University of Rio Grande do Norte Natal Rio Grande do Norte Brazil

**Keywords:** epididymis, rats, reproductive parameters, testis, trazodone

## Abstract

Trazodone is an antidepressant approved for the treatment of major depressive disorder and is associated with a low incidence of sexual dysfunction. However, its effects on male reproductive health are not yet fully understood. Therefore, this study aimed to evaluate the effects of repeated trazodone administration on reproductive parameters and the distal cauda epididymal duct contractions in rats. Adult male Wistar rats were orally treated by gavage for 21 days with saline (control) or trazodone at 10 and 20 mg/kg. After treatment, the blood and reproductive organs were collected for the experimental analysis. Trazodone induced adverse reproductive effects in rats, evidenced by testicular tissue damage, increased intratesticular lipid peroxidation and reduced sperm production and sperm count in different epididymal regions. Additionally, trazodone treatment reduced progressive sperm motility, the number of viable and morphologically normal sperm, indicating decreased sperm quality. The contraction of the isolated distal cauda epididymal duct was also impaired by trazodone treatment, suggesting possible alterations during the emission phase of ejaculation. In conclusion, trazodone exhibited significant reproductive effects that may impair male fertility. These findings provide, to our knowledge, the first integrated assessment of trazodone‐induced changes in testicular morphology, sperm parameters and epididymal duct contractility.

## Plain Language Summary

Trazodone is an antidepressant commonly prescribed for depression and sleep disorders, but little is known about its effects on male fertility. In this study, rats treated with trazodone showed damage to the testes, lower sperm production, poorer sperm quality and impaired contractions of the epididymis, a structure important for sperm storage and ejaculation. These effects were associated with increased oxidative stress in testicular tissue. Our findings suggest that trazodone may negatively affect male reproductive function and highlight the need for further studies to determine whether similar effects may occur in men using this medication.

## Graphical abstract

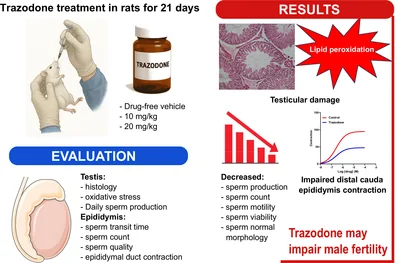

## 1 Introduction

Trazodone is a second‐generation antidepressant widely used for the treatment of major depressive disorder and other neuropsychiatric conditions [1]. Its pharmacological profile involves multimodal actions, including inhibition of the serotonin reuptake transporter (SERT) and antagonism of 5‐HT_2A_/_2C_ serotonin receptors, as well as blockade of H_1_ histamine receptors and α_1_‐adrenoceptors [2, 3].

Despite the broad therapeutic potential of trazodone, concerns regarding its safety profile have been raised, particularly with respect to adverse effects such as sedation, orthostatic hypotension, QT prolongation, priapism and endocrine disturbances such as hyperprolactinemia [1]. Importantly, data on the impact of trazodone on male reproductive health remain limited and inconclusive. Although clinical studies have already documented sexual dysfunctions such as ejaculatory disturbances [4], initial preclinical findings suggest that trazodone may adversely affect sperm quality and testicular structure [5].

Male reproductive capacity can be negatively affected by a wide range of therapeutic agents, including antidepressants, through mechanisms that interfere with the hypothalamic pituitary gonadal (HPG) axis or by directly impairing testicular function [6]. Pharmacological interference with the HPG axis may compromise spermatogenesis and hormonal homeostasis, whereas direct toxic effects on reproductive tissues may induce oxidative stress, apoptosis and cytotoxicity in Sertoli, Leydig and germ cells [5, 6, 7, 8].

The epididymis is also considered an important target of the reproductive effects of different drugs [9]. The spontaneous motor activity of smooth muscle cells in the epididymal duct from caput, corpus and proximal cauda epididymis plays an important role in sperm transport, maturation and storage and is strictly regulated by hormonal, paracrine and autonomic mechanisms. Moreover, the distal cauda region of the epididymis functions not only as a storage reservoir for mature spermatozoa but also contributes to sperm emission during ejaculation through smooth muscle contractions of the epididymal duct mediated by adrenergic neurons in rodents and humans [9, 10]. Dysregulation in epididymal duct motor activity, either through hormonal imbalance or direct neuromodulatory drug effects, can alter sperm transit time and maturation, ultimately affecting sperm quantity and quality. Thus, impaired contractile function in the distal cauda epididymal duct may interfere with ejaculation by disrupting the proper transfer of semen into the posterior urethra [11, 12].

Although some reports suggest that trazodone may increase prolactin levels [6] and alter testosterone production [5], studies systematically investigating its effects on male reproductive function, particularly on epididymal physiology, are lacking. Therefore, a comprehensive investigation connecting the effects of trazodone at multiple levels of the male reproductive tract, from the gonad to the posttesticular duct, remains to be conducted.

To address this gap in knowledge, we hypothesized that repeated trazodone administration would impair male reproductive function by affecting both testicular integrity and epididymal function. Accordingly, we evaluated testicular morphology, testicular and epididymal sperm parameters, and the contractile activity of the distal cauda epididymal duct following in vivo trazodone exposure in Wistar rats, a well‐established model for reproductive biology studies [13, 14]. Our findings may contribute to a better understanding of the male reproductive effects of trazodone and the mechanisms underlying its impact on fertility.

## 2 Materials and Methods

### 2.1 Drugs and Reagents

All drugs were purchased from Sigma Chemical Co. (St. Louis, MO, USA), and all reagents were obtained from Dinâmica (Brazil).

### 2.2 Animals and Treatment

A total of 21 male Wistar rats, 3–4 months old and weighing 300–400 g, were used in this study. The animals were obtained from the Central Animal Facility of the Biosciences Center at the Federal University of Rio Grande do Norte. They were housed in the Animal Facility of the Department of Biophysics and Pharmacology, in standard ventilated cages (four animals per cage), with free access to food (Purina Labina, Brazil) and water. Environmental conditions were maintained under a 12‐h light/dark cycle (lights on from 6:00 am to 6:00 pm) and a controlled temperature of 23°C ± 1°C. Each animal was considered an independent experimental unit. No predefined exclusion criteria were established for animals. Experimental procedures were approved by the Animal Ethics Committee (CEUA) of the Federal University of Rio Grande do Norte (Approval Certificate No. 352.031/2023; Protocol No. 031/2023) and followed the ARRIVE guidelines [15]. In addition, the study was conducted in accordance with the Basic and Clinical Pharmacology and Toxicology policy for experimental and clinical studies [16].

Rats were randomly assigned to experimental groups using a simple randomization procedure based on random number generation and received oral administration (via gavage) of trazodone at 10 or 20 mg/kg once daily for 21 consecutive days. The control group received drug‐free vehicle, administered at the same volume and via the same route as the trazodone‐treated animals. The selected doses and route of administration were based on previous studies investigating the effects of trazodone on reproductive parameters in rats [5]. The 21‐day treatment period was selected to assess early reproductive effects following subacute exposure, considering that previous studies with other pharmacological agents have reported testicular and spermatogenic alterations within 7–21 days of exposure [17, 18], despite the complete spermatogenic cycle in rats lasting approximately 54 days [19]. Furthermore, applying the animal‐to‐human dose conversion described by Nair and Jacob [20], the 10 and 20 mg/kg doses in rats correspond to human‐equivalent doses of 1.62 and 3.24 mg/kg, respectively. For a 60 kg adult, these values translate to approximately 97.2 mg/day and 194.4 mg/day, which fall within the therapeutic range used in adult humans (typically 50–150 mg/day), with the possibility of increasing up to 600 mg/day in hospitalized patients [1]. Moreover, to minimize potential confounding factors, animals from different experimental groups were housed under identical environmental conditions, handled similarly throughout the study, and experimental procedures were conducted under standardized conditions.

Body weight was monitored daily throughout the treatment period for general health and signs of distress. No unexpected adverse events were observed. Humane endpoints were not required because no severe pain or distress conditions were identified during the treatment period. At the end of the treatment period, all animals were euthanized by decapitation 24 h after the last drug administration, and blood samples were collected for serum testosterone analysis. In addition, the reproductive organs (testes, epididymides, prostate and seminal vesicles) were excised, weighed and processed for subsequent experimental procedures, including histopathological analysis, assessment of sperm parameters in the testes and epididymides or isolated organ bath experiments using segments of the distal cauda epididymal duct, as described below. For histological and morphometric analyses, as well as sperm parameter assessments, the evaluator was blinded to the experimental group allocation. Blinding was not applied during drug administration, serum testosterone measurements or functional experiments.

### 2.3 Experimental Design and Sample Allocation

The animals described above were allocated into the following three experimental groups (*n* = 7/group): control, trazodone 10 mg/kg and trazodone 20 mg/kg. Blood samples were collected from all animals for serum testosterone analysis (*n* = 7/group). Histological and oxidative stress analyses were performed using five animals per group because of limitations in reagent availability and laboratory resources. Quantitative sperm analyses were conducted with six animals per group because samples from one animal in each group could not be analysed owing to technical issues during sample processing. Qualitative sperm analyses were performed using samples from seven control animals, five animals in the trazodone 10 mg/kg group, and seven animals in the trazodone 20 mg/kg group because the quality of the sperm smears obtained from two animals in the trazodone 10 mg/kg group was insufficient for reliable morphological evaluation. Epididymal duct contractility was assessed in seven control animals, seven animals in the trazodone 10 mg/kg group and six animals in the trazodone 20 mg/kg group. One epididymal preparation from the trazodone 20 mg/kg group was damaged during tissue isolation and was therefore excluded from the analysis.

### 2.4 Serum Testosterone Analysis

Blood samples collected from treated and control animals were centrifuged to obtain serum, which was stored at freezing temperatures until hormone analyses were performed. Serum total testosterone concentrations were determined using the VITROS MicroWell Testosterone immunoassay (Reagent Pack, Catalogue No. 1435205; Ortho Clinical Diagnostics, Raritan, NJ, USA) following the manufacturer's protocol on the VITROS 5600 Immunodiagnostic System. The assay is based on a competitive chemiluminescent immunoassay and is also compatible with the VITROS ECi/ECiQ, 3600 and XT 7600 Immunodiagnostic Systems. According to the manufacturer, the analytical measurement range extends from 4.90 to 2160 ng/dL, with intra‐assay precision ranging from 1.1% to 3.6% and within‐laboratory (interassay) precision ranging from 3.1% to 5.1%. This immunoassay has been previously applied for the determination of serum testosterone concentrations in rats [21]. This analysis aimed to determine whether the observed alterations were related to changes in sex hormone levels or to direct effects of trazodone on the testis or epididymis.

### 2.5 Histological Analysis of the Testis

The testes were excised from both treated and control animals, cleaned of surrounding connective tissue and weighed. The major and minor axes were measured using a digital calliper. Testicular volume was estimated using Scherle's method [22, 23], according to the formula: volume (mm^3^) = density × mass. After weighing, the testes were fixed in Bouin's solution for 24 h. Subsequently, the samples were washed, preserved in 70% ethanol, dehydrated in ascending concentrations of ethanol, cleared in xylene and embedded in paraffin. Sections of 3‐μm thickness were obtained using a microtome. These sections were mounted on glass slides and stained with haematoxylin and eosin (H&E) for morphological analysis under a light microscope, using the NIS‐Elements Advanced Research imaging system (Nikon Corporation, Tokyo, Japan).

For histopathological evaluation, 100 seminiferous tubules per animal, preferably with a circular cross‐section, were analysed to ensure accuracy in the measurements of seminiferous tubule diameter (μm), epithelial height (μm) and lumen diameter (μm). All measurements were performed using ImageJ software (National Institutes of Health, USA), calibrated with a stage micrometre. The evaluator was blinded to the experimental groups during the analysis.

The numbers of Sertoli and Leydig cells were also quantified in 10 cross‐sections of seminiferous tubules per animal, preferentially selecting tubules with a circular profile. Their numerical densities were calculated based on histological cross‐sections stained with H&E. Digital images were acquired and morphometric analyses were carried out using ImageJ. To estimate the number of Sertoli cells per mm^2^ of seminiferous tubule (Se/mm^2^), the following formula was used: Se/mm^2^ = *N*_s_/*A*_t_, where *N*_s_ is the total number of Sertoli cells counted within the seminiferous tubules, and *A*_t_ is the total tubular area analysed (in mm^2^). Similarly, the number of Leydig cells per mm^2^ of interstitial tissue (Le/mm^2^) was determined using: Le/mm^2^ = *N*_l_/*A*_i_, where *N*_l_ represents the number of Leydig cells counted in the interstitial space, and *A*ᵢ is the total area of interstitial tissue analysed (in mm^2^). To improve accuracy, the analysed areas were manually delineated in randomly selected microscopic fields using a calibrated grid. Cell counts were corrected using the method originally described by Abercrombie [24] and later modified by Amann and Almquist [25] to compensate for overestimation due to section thickness and nuclear diameter: $N_{\text{real}}=N_{\text{observed}}\;x\;\frac{T}{T+\sqrt{{\left(\frac{D}{2}\right)}^{2}-{\left(\frac{D}{4}\right)}^{2}}}$, where *T* is the section thickness (μm), and *D* is the mean nuclear diameter of the cells being quantified. Finally, a qualitative assessment of the seminiferous tubules was also performed.

### 2.6 Evaluation of Testicular Oxidative Stress

To evaluate oxidative stress in the testes of control and trazodone‐treated animals, malondialdehyde (MDA) and reduced glutathione (GSH) levels were measured. MDA is a marker of lipid peroxidation and a secondary product generated during oxidative degradation of lipids. MDA levels were determined by its reaction with thiobarbituric acid (TBA), forming MDA–TBA2 complexes that exhibit a pinkish‐red colour and can be measured spectrophotometrically [26]. For this assay, testicular parenchyma samples from control and treated animals were homogenized in phosphate‐buffered saline (PBS 0.01 M, pH 6.5) containing protease inhibitors. The homogenate was then centrifuged, and the resulting supernatant was mixed with 10% phosphotungstic acid to precipitate proteins. After a second centrifugation step, the supernatant was combined with 50 mM butylated hydroxytoluene (BHT) and 0.67% thiobarbituric acid. The mixture was heated to approximately 95°C for 60 min to allow formation of the MDA–TBA2 complex. After cooling, absorbance was measured at 535 nm, and MDA concentration was calculated based on a standard curve.

The reaction of GSH with 5,5′‐dithiobis‐(2‐nitrobenzoic acid) (DTNB) generates oxidized glutathione (GSSG) and 2‐nitro‐5‐thiobenzoic acid (TNB). The amount of TNB produced is proportional to the GSH concentration in the sample and produces a yellow colour measurable by spectrophotometry. For GSH quantification, testicular parenchyma samples were homogenized in 0.02 M EDTA buffer. Proteins were then precipitated using 50% trichloroacetic acid, followed by centrifugation to collect the supernatant. A portion of the supernatant was mixed with 0.4 M Tris‐EDTA buffer (pH 8.9), and 1 mM DTNB was added. The reaction was incubated at 37°C for 10 min, and absorbance was measured at 412 nm. GSH concentration was determined using a standard curve. MDA and GSH concentrations were normalized based on total protein content in the testicular parenchyma, as determined by the Lowry method.

### 2.7 Determination of Daily Sperm Production, Epididymal Sperm Reserves and Sperm Transit Time

The testes and epididymides from treated and control animals were processed to evaluate daily sperm production, sperm transit time and sperm concentration. For the assessment of daily sperm production, the testes were isolated, decapsulated and weighed. Homogenization‐resistant spermatids at stage 19 of spermiogenesis were quantified following previously described methods [27, 28, 29]. Each decapsulated testis was initially homogenized in 5 mL of 0.9% NaCl containing 0.5% Triton X‐100, followed by sonication for 30 s. After a tenfold dilution, a sample was loaded into a Neubauer counting chamber, and spermatids were counted. Four samples were evaluated per animal. Daily sperm production was assessed by dividing the number of stage 19 spermatids per testis (number of spermatids × 10^6^/testis) by 6.1, which corresponds to the duration (in days) these spermatids remain in the seminiferous epithelium.

In animals treated with trazodone at either dose or with vehicle, the epididymides were carefully dissected and segmented into caput/corpus (encompassing segments 1–14, as described by Domeniconi et al. [30]) and cauda (segments 15–19). Each region was weighed and subsequently minced into small fragments. Tissue homogenization was performed mechanically using a solution composed of 0.9% NaCl and 0.5% Triton X‐100. The volume of solution was determined according to tissue mass: 1 mL per 200 mg of the caput/corpus segment and 1 mL per 100 mg of the cauda segment. The homogenates were subsequently diluted 1:20, and aliquots were loaded into Neubauer counting chambers to quantify sperm cells. For each animal, four independent chamber counts were conducted. Sperm transit time through the epididymal regions was estimated by dividing the total number of sperm (expressed as ×10^6^ per organ) by the daily sperm production.

### 2.8 Sperm Motility, Vitality and Morphology Analysis

Epididymal fluid samples from treated and control animals were collected by making small incisions (< 1 mm) in segment 19 of the cauda epididymis region [30]. For sperm motility analysis, samples were resuspended in 1 mL of phosphate‐buffered saline (PBS; 137 mM NaCl, 2.7 mM KCl, 10 mM Na_2_HPO_4_, 1 mM KH_2_PO_4_, pH 7.4) and examined under a light microscope. A total of 200 spermatozoa were evaluated per sample, assessing total motility (%), progressive motility (%) and nonprogressive motility (%). For sperm vitality assessment, epididymal fluid samples were collected as described above, then diluted 1:1 with 5% eosin yellow dye. The final mixture was homogenized, applied onto a microscope slide, covered with a coverslip and examined microscopically. Two hundred intact spermatozoa were observed and classified as either live or dead, with results expressed as the percentage of viable sperm. Sperm morphology was evaluated using aliquots of the epididymal fluid samples obtained as above. A 20 μL aliquot was spread on a microscope slide to prepare a smear, and 200 spermatozoa were examined under light microscopy. Spermatozoa were classified as morphologically normal or abnormal. Morphologically abnormal spermatozoa were further categorized according to the type of defect, including head, midpiece or tail abnormalities.

### 2.9 Functional Experiments in Isolated Organ Bath

Segments of approximately 1.5 cm from the distal cauda epididymal duct (segment 19 [30]) were isolated from treated and control animals and mounted under an initial resting tension of 1.0 g in an isolated organ bath containing aerated nutritive solution (in mM: 138.0 NaCl, 5.7 KCl, 1.8 CaCl_2_.2H_2_O, 15.0 NaHCO_3_, 0.36 NaH_2_PO_4_.H_2_O and 5.5 glucose) at 32°C (pH 7.35–7.40). One end of each tissue segment was fixed to a glass hook, and the other was attached to an isometric force transducer connected to a physiograph for contraction recordings. Following tissue mounting, a 30‐min stabilization period was allowed. Tissue viability was then assessed by incubating the preparation with 80 mM KCl for 5 min. The tissue was then thoroughly washed with the nutritive solution. After a second 30‐min stabilization period, isometric contractions were induced by cumulative concentration–response curves for agonists (noradrenaline or carbachol) or by a single administration of 80 mM KCl for 5 min. Pharmacological parameters, including the maximum response (*E*_max_, indicating efficacy) and the negative logarithm of the concentration that produces 50% of the maximal response (‐logEC_50_, indicating potency) were calculated and compared among experimental groups. Detailed protocols for these methods are available in previous publications by our group [27, 31, 32].

### 2.10 Data Analysis

Isometric contractions were measured and expressed as grams of tension (g). Cumulative concentration‐response curves (CCRCs) were constructed by nonlinear regression analysis to determine pharmacological parameters, including ‐logEC_50_ and *E*_max_, using GraphPad Prism Version 8 (San Diego, CA, USA). Data are presented as mean ± standard error of the mean (SEM). Data normality was assessed using the Shapiro–Wilk test, and all datasets showed a normal distribution. One‐way analysis of variance (ANOVA) followed by Dunnett's test was used for comparisons. A significance level of *p* < 0.05 was adopted. The number of biological replicates used in each analysis is described in the *Experimental Design and Sample Allocation* subsection. Each biological replicate consisted of tissue or blood collected from a different animal. Sample sizes were based on previous studies from our group evaluating reproductive and epididymal parameters in rats treated with pharmacological agents and on our previous experience with the variability of these experimental endpoints [27, 31, 32].

## 3 Results

### 3.1 Evaluation of Body and Reproductive Organ Weights

Oral administration of trazodone at 10 or 20 mg/kg for 21 days did not affect body weight at the end of the treatment period (Table 1). The absolute weights of the testis and seminal vesicle were increased in animals treated with 10 and 20 mg/kg trazodone, respectively, compared with the control group. Analysis of the relative reproductive organ weights showed a significant increase only in the seminal vesicle of animals treated with 20 mg/kg trazodone (Table 1).

**TABLE 1:** Body weight and reproductive organ weights of rats treated with trazodone (10 or 20 mg/kg) or drug‐free vehicle (control) for 21 days.

Parameters	Experimental groups		
	Control (*n* = 7)	Trazodone 10 mg/kg (*n* = 7)	Trazodone 20 mg/kg (*n* = 7)
**Body weight (g)**			
Start of treatment	426.7 ± 10.8	440.1 ± 12.8	414.7 ± 9.7
End of treatment	454.7 ± 14.3	473.7 ± 14.7	443.7 ± 10.5
Weight change	28.0 ± 4.6	33.6 ± 4.2	29.0 ± 3.7
**Absolute reproductive organ weight (g)**			
Testis	1.78 ± 0.05	1.95 ± 0.04^a^	1.75 ± 0.03
Empty seminal vesicle	0.50 ± 0.06	0.59 ± 0.07	0.73 ± 0.07^a^
Vas deferens	0.09 ± 0.006	0.09 ± 0.006	0.09 ± 0.009
Prostate	0.29 ± 0.04	0.31 ± 0.02	0.36 ± 0.03
Epididymis	0.62 ± 0.02	0.67 ± 0.02	0.62 ± 0.01
**Relative reproductive organ weight (g tissue/100 g animal)**			
Testis	0.39 ± 0.005	0.41 ± 0.009	0.39 ± 0.012
Empty seminal vesicle	0.11 ± 0.01	0.12 ± 0.01	0.16 ± 0.01^a^
Vas deferens	0.02 ± 0.001	0.02 ± 0.001	0.02 ± 0.002
Prostate	0.063 ± 0.009	0.067 ± 0.005	0.083 ± 0.008
Epididymis	0.14 ± 0.004	0.14 ± 0.005	0.14 ± 0.005

*Note:* Values are expressed as mean ± SEM n: sample size. Weight change corresponds to the difference between initial and final body weight.

Abbreviation: *n* = sample size.

^a^ *p* < 0.05 compared with the control group.

### 3.2 Analysis of Serum Testosterone Levels

**FIGURE 1:**
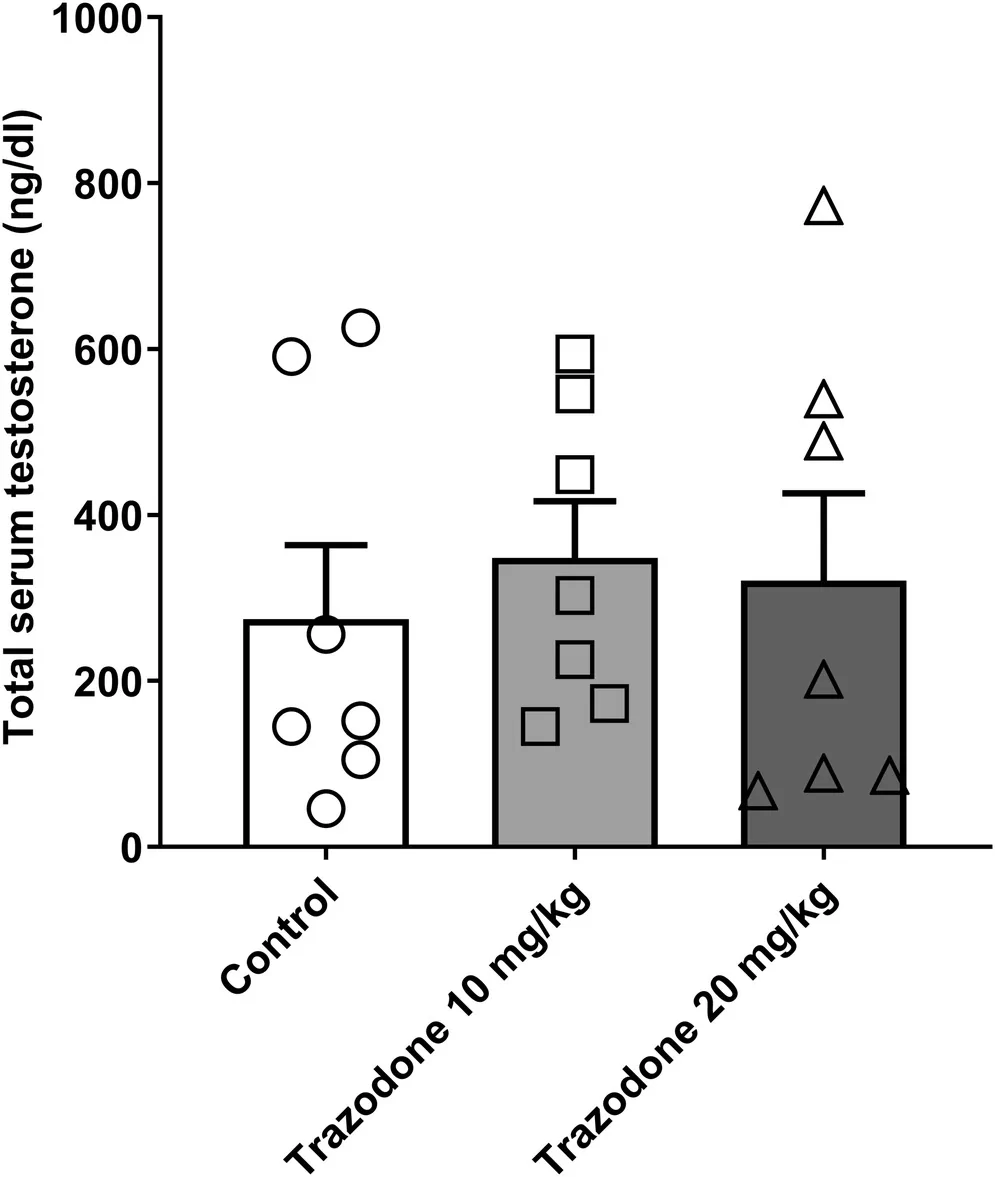
Bar graph showing the serum testosterone levels in animals treated with trazodone (10 or 20 mg/kg) or drug‐free vehicle (control) for 21 days. Each bar shows the mean ± SEM of seven samples. Circles indicate individual values for each sample within the respective group.

Treatment with trazodone at 10 or 20 mg/kg for 21 days did not significantly alter serum testosterone levels compared with the control group (Figure 1).

### 3.3 Histological Evaluation of the Testes From Rats Treated With Trazodone

**FIGURE 2:**
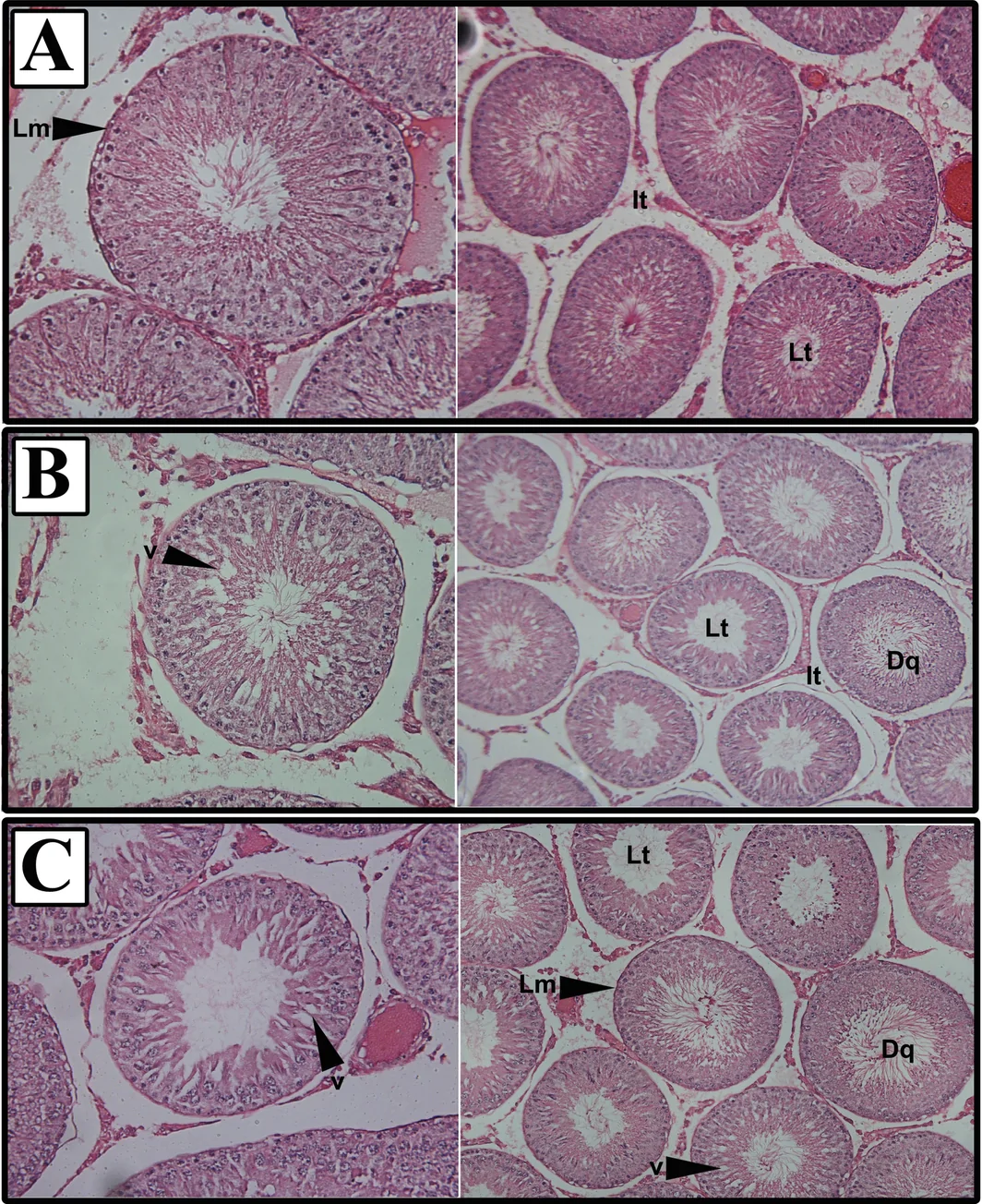
Photomicrograph of tubular sections of the testes of rats from the control group (A) and treated with trazodone 10 mg/kg (B) or 20 mg/kg (C) for 21 days. HE staining, 20× (left) or 10× (right) magnification. Cell debris from the desquamation of the seminiferous epithelium (Dq), interstitial tissue (It), limiting lamina (Lm), tubular lumen (Lt) and vacuolization (V).

We evaluated the effects of 21‐day treatment with trazodone on the testicular histology of rats through quantitative (Table 2) and qualitative histological analysis (Figure 2). Animals treated with trazodone at 20 mg/kg showed a significant reduction in the absolute testicular volume compared with control animals (Table 2). In contrast, no significant changes were observed in the diameter of the seminiferous tubules in trazodone‐treated animals (Table 2). However, there was an approximately 20% reduction in tubular epithelium height in animals treated with both trazodone doses, which was accompanied by an increase in luminal area diameter of the seminiferous tubules of approximately 40% for trazodone 10 mg/kg and 55% for trazodone 20 mg/kg (Table 2). The numerical densities of Leydig and Sertoli cells were significantly reduced in animals treated for 21 days with trazodone at 10 mg/kg (~30% and ~50%, respectively) and 20 mg/kg (~50% and ~63%, respectively) (Table 2).

**TABLE 2:** Quantitative histological analysis of the testes from rats treated with trazodone (10 or 20 mg/kg) or drug‐free vehicle (control) for 21 days.

Parameters	Experimental groups		
	Control (*n* = 5)	Trazodone 10 mg/kg (*n* = 5)	Trazodone 20 mg/kg (*n* = 5)
Absolute testicular volume (mm^3^)	800.5 ± 69.8	654.5 ± 102.2	346.6 ± 39.59^a^
Seminiferous tubule diameter (μm)	347.5 ± 15.5	305.5 ± 17.0	321.1 ± 12.5
Tubular epithelium height (μm)	128.0 ± 5.11	104.5 ± 9.6^a^	102.1 ± 3.07^a^
Lumen area diameter (μm)	78.8 ± 4.0	108.1 ± 11.1^a^	121.1 ± 7.7^a^
Sertoli cells/mm^2^	6.1 ± 1.3	3.0 ± 0.24^a^	2.2 ± 0.7^a^
Leydig cells/mm^2^	13.9 ± 1.0	9.4 ± 1.0^a^	6.9 ± 0.94^a^

*Note:* Values are expressed as mean ± SEM.

Abbreviation: *n* = sample size.

^a^ *p* < 0.05 compared with the control group.

Qualitative histological analysis of testicular tissue from control animals showed preserved and organized seminiferous tubules, with a germinal epithelium displaying well‐defined, layered cellular organization, indicating a normal spermatogenesis process (Figure 2A). In animals treated with trazodone 10 mg/kg, the seminiferous tubules exhibited an increased tubular lumen and decreased cell density in the germinal epithelium, with clear signs of vacuolization and desquamation of the seminiferous epithelium, indicating a possible process of cellular degeneration (Figure 2B). The seminiferous tubules of animals treated with trazodone at 20 mg/kg showed more severe alterations. The lumen was enlarged, and the germinal epithelium appeared more atrophied, with fewer, disorganized cell layers and marked vacuolization and desquamation of the seminiferous epithelium (Figure 2C).

The attachment of the germinal epithelium to the basement membrane was generally preserved in all experimental groups. However, occasional focal areas suggestive of epithelial detachment were observed in some seminiferous tubules, particularly in the trazodone 10 mg/kg group (Figure 2B‐right). As this feature was not consistently observed across the examined cross‐sections and may represent a histological artefact, no definitive conclusion can be drawn regarding its biological significance. In addition, no apparent morphological alterations were observed in the interstitial blood vessels of trazodone‐treated animals compared with the control group, and the vascular architecture appeared preserved in all experimental groups.

### 3.4 Determination of Oxidative Stress in the Testes of Rats Treated With Trazodone

**FIGURE 3:**
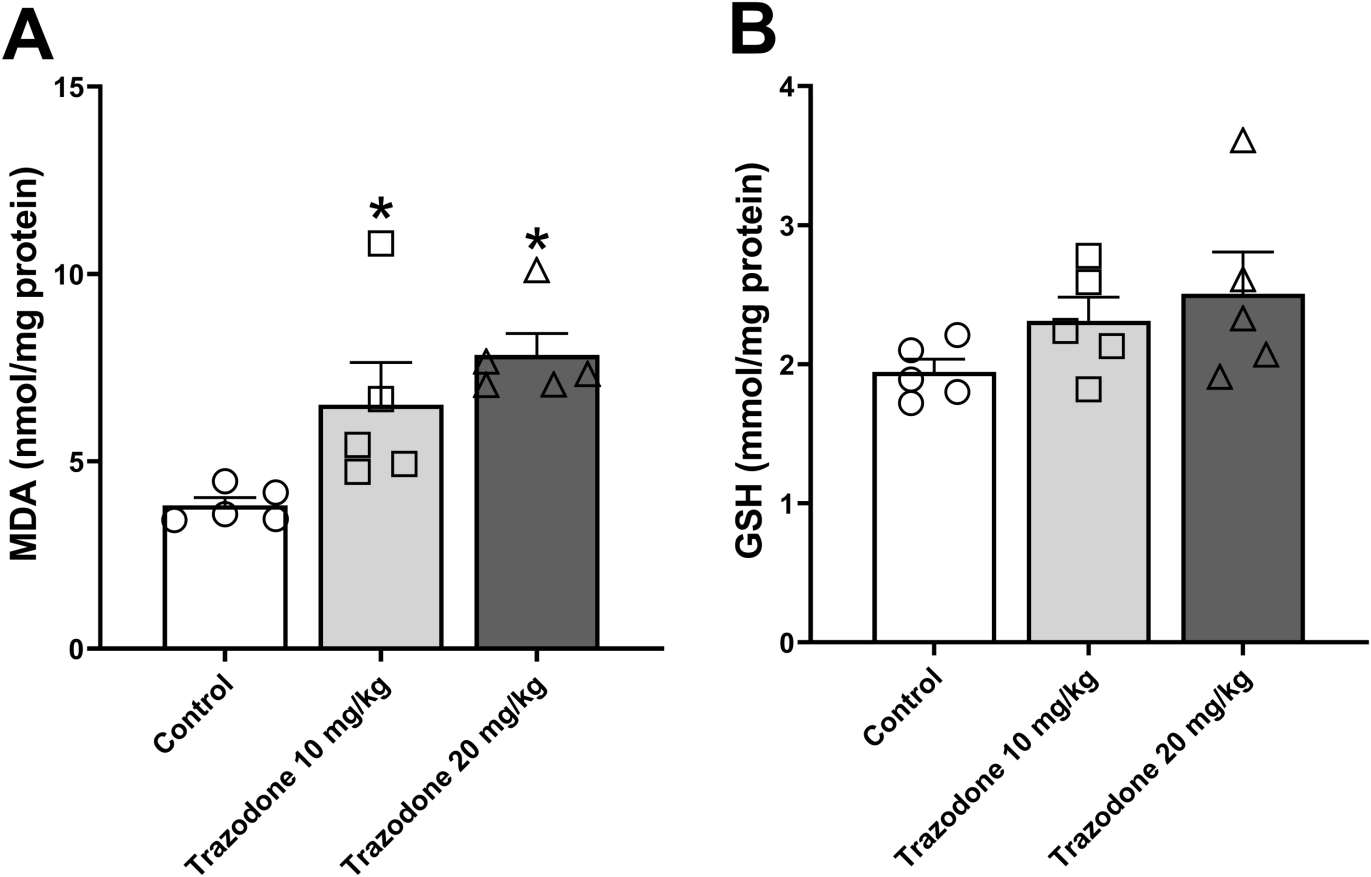
Malondialdehyde (MDA) (A) and reduced glutathione (GSH) levels (B) in the testes of rats treated with trazodone (10 or 20 mg/kg) or drug‐free vehicle (control) for 21 days. Each bar represents the mean ± SEM of five samples. Symbols indicate individual values for each sample within each group. *p < 0.05 compared with the control group.

Oxidative stress was assessed by measuring the levels of MDA and reduced GSH in the testes of animals treated with trazodone (10 and 20 mg/kg) or drug‐free vehicle (control) for 21 days. Trazodone treatment at both doses significantly increased testicular MDA levels compared with the control group (Figure 3A). On the other hand, GSH levels in the testes were not altered in animals treated with trazodone at either dose (Figure 3B).

### 3.5 Effects of Trazodone Treatment on Sperm Parameters

The number of spermatids and daily sperm production were significantly reduced after 21 days of treatment with trazodone at both 10 and 20 mg/kg doses (Table 3). Treatment with 20 mg/kg trazodone, but not 10 mg/kg, led to a reduction in sperm count in the caput/corpus region of the epididymis (Table 3). However, trazodone treatment at both doses significantly decreased sperm counts in the cauda region of the epididymis (Table 3). Conversely, trazodone treatment at both doses for 21 days did not alter sperm transit time in either the caput/corpus or cauda regions of the epididymis (Table 3).

**TABLE 3:** Quantitative sperm parameters in rats submitted to the treatment with trazodone (10 or 20 mg/kg) or drug‐free vehicle (control) for 21 days.

Sperm parameters	Experimental groups		
	Control (*n* = 6)	Trazodone 10 mg/kg (*n* = 6)	Trazodone 20 mg/kg (*n* = 6)
**Testis**			
Spermatid count, × 10^6^/testis	214.2 ± 6.0	176.4 ± 10.4^a^	170.9 ± 10.1^a^
Spermatid count, × 10^6^/g/testis	156.4 ± 5.0	111.1 ± 6.0^a^	115.9 ± 6.5^a^
Daily sperm production, ×10^6^/testis/day	35.1 ± 1.0	28.9 ± 1.7^a^	28.0 ± 1.6^a^
Daily sperm production, ×10^6^/g testis/day	25.6 ± 0.8	18.2 ± 1.0^a^	19.0 ± 1.0^a^
**Caput/corpus of the epididymis**			
Sperm count, ×10^6^/organ	117.4 ± 13.0	102.5 ± 6.8	85.76 ± 7.6^a^
Sperm count, ×10^6^/g/organ	302.7 ± 13.4	253.5 ± 7.9	231.9 ± 23.0^a^
Transit time (days)	3.9 ± 0.4	3.6 ± 0.1	3.5 ± 0.5
**Cauda of the epididymis**			
Sperm count, ×10^6^/organ	135.3 ± 7.9	110.4 ± 10.4	101.4 ± 6.2^a^
Sperm count, ×10^6^/g/organ	723.1 ± 32.8	547.8 ± 45.3^a^	579.9 ± 40.9^a^
Transit time (days)	4.3 ± 0.5	3.9 ± 0.2	3.9 ± 0.3

*Note:* Values are expressed as mean ± SEM.

Abbreviation: *n* = sample size.

^a^ *p* < 0.05 compared with the control group.

**FIGURE 4:**
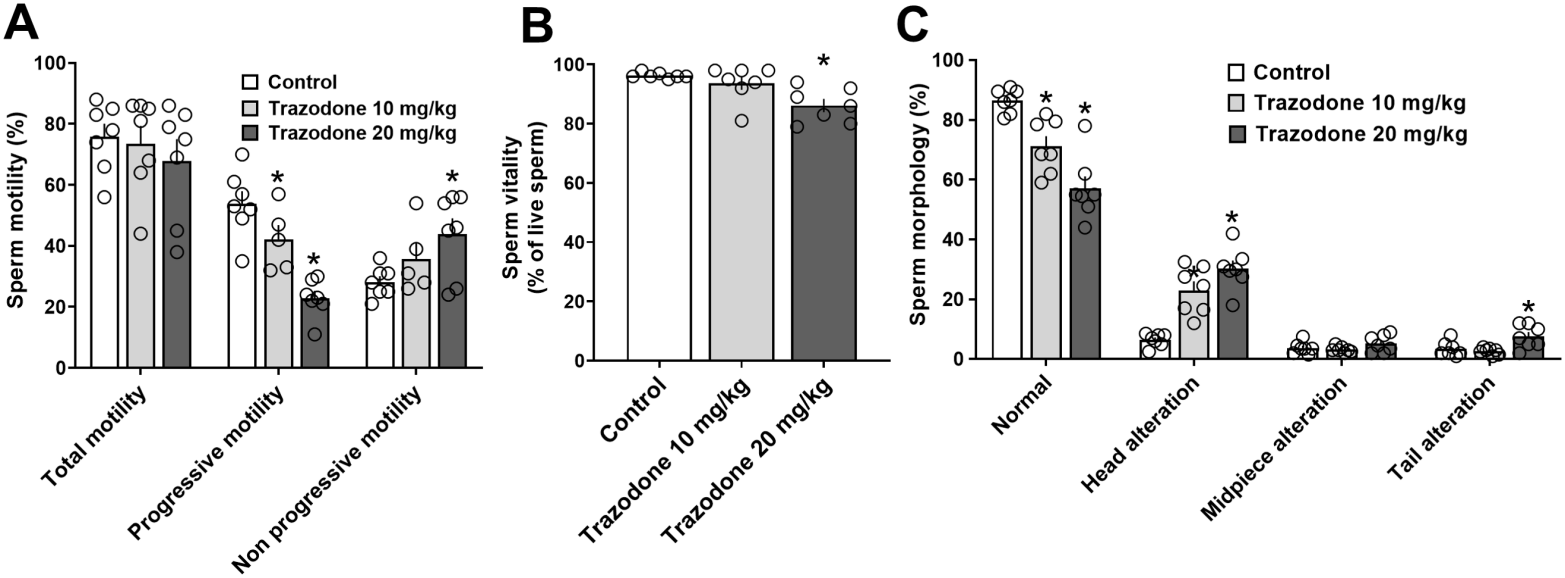
Evaluation of sperm motility (A), viability (B) and morphology (C) in samples collected from the distal cauda of the epididymis from rats treated with trazodone (10 or 20 mg/kg) or drug‐free vehicle (control) for 21 days. Each bar represents the mean ± SEM of five to seven samples. Symbols indicate individual values for each sample within the respective group. *p < 0.05 compared with the control.

Trazodone treatment for 21 days was also able to alter qualitative sperm parameters in rats (Figure 4). Treatment with trazodone at 10 and 20 mg/kg significantly reduced progressive sperm motility by approximately 22% and 58%, respectively, in samples collected from the distal cauda of the epididymis (Figure 4A). Additionally, an approximately 56% increase in nonprogressive motility was observed in sperm collected from the distal cauda of rats treated with trazodone 20 mg/kg for 21 days (Figure 4A). Regarding sperm viability, a mild reduction (~10%) was detected in samples from the distal cauda of rats treated with 20 mg/kg trazodone compared with controls (Figure 4B). Furthermore, spermatozoa with head abnormalities accounted for 23% and 30% of all evaluated spermatozoa in the trazodone 10 and 20 mg/kg groups, respectively, compared with 6% in the control group (Figure 4C). In addition, the percentage of spermatozoa with tail defects was higher in the 20 mg/kg group (7.6% ± 1.4%, *n* = 7) than in the control group (3.4 ± 0.9%, *n* = 7) (Figure 4C).

### 3.6 Effects of Trazodone Treatment on Contractions of the Distal Cauda Epididymal Duct in Rats

**FIGURE 5:**
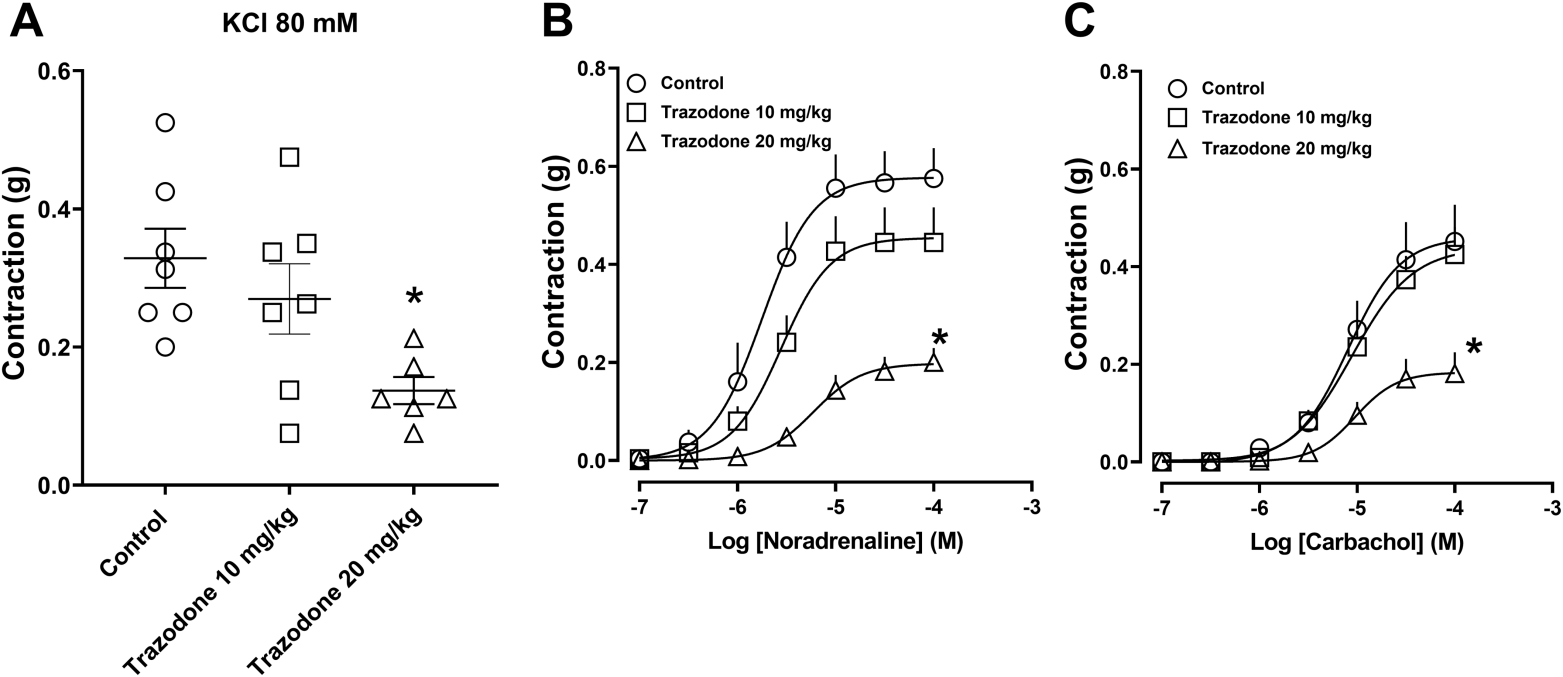
Contractions of the isolated epididymal duct from the distal cauda epididymis induced by a single concentration of KCl (80 mM for 5 min) (A) or by cumulative concentrations of noradrenaline (B) or carbachol (C) in rats treated with trazodone (10 or 20 mg/kg) or drug‐free vehicle (control). Panel A shows the mean ± SEM of six to seven experiments. Each point in panels B and C represents the mean ± SEM of six to seven experiments. *p < 0.05 compared with the control.

The effects of 21‐day trazodone treatment were also evaluated on contractions of the epididymal duct isolated from distal cauda epididymis induced by the depolarizing agent KCl or by agonists such as noradrenaline (an adrenergic receptor agonist) and carbachol (a muscarinic acetylcholine receptor agonist). Only trazodone treatment at 20 mg/kg significantly affected the contractile activity of the isolated distal cauda epididymal duct (Figure 5). Specifically, treatment with trazodone 20 mg/kg reduced KCl‐induced contractions by approximately 60% (Figure 5A and Table 4). Trazodone at this dose also reduced the maximal response (*E*_max_) to noradrenaline and carbachol by 65% and 60%, respectively (Figure 5B,C and Table 4). The potency of noradrenaline, but not carbachol, was decreased by approximately fourfold in the isolated distal cauda epididymal duct of rats treated with trazodone 20 mg/kg for 21 days (Figure 5B and Table 4).

**TABLE 4:** Maximum effect (*E*_max_) and potency (‐LogEC_50_) values for noradrenaline or carbachol in epididymal duct isolated from the distal cauda epididymis of rats treated with trazodone (10 or 20 mg/kg) or drug‐free vehicle (control).

Noradrenaline	Pharmacological parameters	
	*E*_max_	‐LogEC_50_
Control (*n* = 7)	0.57 ± 0.06	5.7 ± 0.14
Trazodone 10 mg/kg (*n* = 7)	0.44 ± 0.07	5.6 ± 0.06
Trazodone 20 mg/kg (*n* = 6)	0.20 ± 0.02^a^	5.1 ± 0.13^a^
**Carbachol**		
Control (*n* = 7)	0.45 ± 0.07	5.0 ± 0.09
Trazodone 10 mg/kg (*n* = 7)	0.42 ± 0.05	5.0 ± 0.13
Trazodone 20 mg/kg (*n* = 6)	0.18 ± 0.05^a^	5.0 ± 0.07

*Note:* Values are expressed as mean ± SEM.

Abbreviation: *n* = sample size.

^a^ *p* < 0.05 compared with the control group.

## 4 Discussion

Unlike most antidepressants, trazodone is known to have a low propensity to impair libido [33]. Moreover, it has been reported that this drug may facilitate penile erection by blocking peripheral α_1_‐adrenoceptors and through an as yet unknown central mechanism [34]. However, although trazodone does not affect libido and may exert favourable effects on erection, its impact on testicular function, sperm parameters and the motor activity of the epididymal duct is not fully understood. In this study, we found that repeated in vivo treatment with trazodone induced adverse reproductive effects in male rats. These effects were characterized by testicular damage, reduced sperm production, decreased sperm count and quality, as well as alterations in the contractility of the isolated distal cauda epididymal duct.

Assessment of body and reproductive organ mass is an important general health indicator and may reveal potential reproductive toxicity induced by drugs [35]. However, no significant decreases were observed in body weight or in the relative weights of most reproductive organs in animals treated with trazodone. Although decreases in organ weight are considered potential indicators of reproductive toxicity, their absence does not rule out alterations in more sensitive markers, such as sperm count, motility and morphology [35, 36]. Interestingly, a significant increase in the relative weight of the empty seminal vesicles was observed in animals treated with the higher dose of trazodone. Although the biological significance of this finding remains unclear, it may indicate a potential effect of trazodone on accessory reproductive organs. However, additional histological and molecular analyses are required to determine whether this alteration is associated with structural remodelling or specific signalling pathways.

Treatment with trazodone reduced daily sperm production in rats, indicating impaired spermatogenesis. Similar effects have been observed with other antidepressants, such as fluoxetine and sertraline, which also decreased daily sperm production, possibly due to reduced testosterone levels [31]. Likewise, compounds such as acetylsalicylic acid, bisphenol A, dipyrone or dexamethasone can interfere with testosterone synthesis or activity, compromising spermatogenesis and reducing sperm output in rodents [6, 32, 37, 38, 39]. However, under our experimental conditions, trazodone did not significantly alter serum testosterone levels.

Studies investigating the effects of trazodone on spermatogenesis and testosterone levels have reported conflicting results. Ilgin et al. [5] demonstrated that rats treated with trazodone (5–20 mg/kg for 28 days) showed spermatogenic impairment accompanied by increased levels of testosterone, LH and FSH. In contrast, Khedr and Werida [40] reported that treatment with trazodone 20 mg/kg for 28 days reduced testosterone, FSH and LH levels and impaired spermatogenesis. Although reduced testosterone is a well‐recognized cause of impaired spermatogenesis [6], our data suggest that decreased daily sperm production may result from direct and/or indirect deleterious effects of trazodone on germ cells, Sertoli cells and/or Leydig cells.

Previous studies using semiquantitative scoring methods have shown that trazodone treatment causes damage to the germinal epithelium and to Sertoli and Leydig cells [5, 40]. Consistent with these findings, our qualitative and quantitative histological analysis showed that trazodone produced similar testicular damage in rats. Although the exact mechanisms underlying trazodone‐induced testicular damage remain unclear, CYP3A4‐mediated metabolism of trazodone may generate reactive metabolites, such as quinoneimine or epoxide species, potentially contributing to oxidative stress, inflammation and testicular degeneration [40]. In fact, we observed elevated levels of testicular MDA in rats treated with trazodone, indicating lipid peroxidation, the most important form of oxidative stress‐related damage in biological systems [41].

The serotonergic system may also contribute to trazodone‐induced testicular damage. Serotonin receptors and SERT are expressed in Sertoli, Leydig and germ cells, and previous studies have shown that serotonergic modulation can impair spermatogenesis and promote oxidative stress and inflammation in the testis [41, 42, 43, 44]. Therefore, trazodone‐mediated SERT inhibition could increase intratesticular serotonin availability and potentially contribute to the testicular alterations observed in the present study.

In contrast, activation of 5‐HT_2A_ receptors in Leydig cells has been associated with reduced testosterone production [42, 44]. Because trazodone acts as a 5‐HT_2_ receptor antagonist [1], this mechanism may help preserve steroidogenic activity and maintain normal testosterone levels despite testicular damage. Additionally, increased LH secretion induced by trazodone, as suggested in previous studies [5], may also contribute to sustaining testosterone production.

The reduction in daily sperm production consequently leads to a decreased sperm number in different regions of the epididymis [31, 32]. It is worth noting that trazodone treatment was able to reduce the sperm count in both the caput/corpus and cauda regions of the epididymis. Another known factor that may contribute to the low sperm count is the motor activity of the epididymal duct, which regulates sperm transit and the number of gametes in the different segments of the epididymis [9]. Therefore, it is essential to assess the effects of trazodone on epididymal duct motor activity by quantifying sperm transit time.

Sperm transit through the epididymis depends on spontaneous and autonomic nervous system‐mediated contractions of the epididymal duct smooth muscle [9, 12, 45]. Given the multitarget pharmacological profile of trazodone, we hypothesized that this drug could alter spontaneous motor activity of the epididymal duct and increase sperm transit time. However, trazodone did not significantly affect sperm transit time, suggesting that the reduced sperm number observed in the epididymis is primarily associated with impaired sperm production.

In contrast, the epididymal duct in the distal cauda region of the epididymis does not exhibit spontaneous motor activity. In this segment, sperm remain stored until ejaculation [9, 14, 45]. During the emission phase of ejaculation, contractions of the distal cauda epididymal duct promote sperm transport into the vas deferens, from where the germ cells are propelled into the prostatic urethra and subsequently ejaculated [14]. These contractions are dependent on autonomic neurotransmitters, particularly noradrenaline acting via α_1A_‐adrenoceptors [14, 46]. Trazodone was able to affect the contractile activity of the distal cauda epididymal duct in rats. At a dose of 20 mg/kg (but not 10 mg/kg), trazodone reduced the contractions induced by KCl and the maximal responses to noradrenaline and carbachol, possibly through inhibition of voltage‐dependent calcium channels [47]. Furthermore, trazodone decreased the potency of noradrenaline, an effect possibly associated with α_1A_‐adrenoceptor antagonism [48]. It is important to highlight that the contractions of the vas deferens, seminal vesicles and prostate during ejaculation also depend on noradrenaline‐mediated activation of α_1A_‐adrenoceptors [49]. In this context, considering the antiadrenergic properties of trazodone, it is plausible to expect that this drug may impair the ejaculatory process. Supporting this hypothesis, a case report described trazodone‐associated ejaculatory inhibition in men [50].

The quality of sperm collected from the cauda epididymis was also investigated, and we found that trazodone treatment reduced progressive motility, the number of viable sperm and the proportion of morphologically normal sperm. The reduction in morphologically normal spermatozoa may reflect disturbances occurring during spermatogenesis and/or epididymal sperm maturation. Sperm morphology is established through the complex process of spermiogenesis, and disturbances in this process can result in abnormal sperm morphology and impaired sperm function [51]. In addition, sperm maturation during epididymal transit is essential for the acquisition of progressive motility and fertilizing capacity [52, 53]. During this process, spermatozoa undergo sequential biochemical and structural modifications as they interact with the specialized epididymal luminal environment [52, 53]. Thus, alterations in epididymal function may contribute to the impaired sperm quality observed in the present study. The composition of the luminal environment of the epididymal duct is also essential for sperm maturation [52]. In this context, serotonergic signalling has been implicated in the regulation of epididymal epithelial secretion and luminal composition [54, 55]. Thus, trazodone‐induced modulation of the epididymal serotonergic system may impair sperm maturation by altering the luminal microenvironment, thereby contributing to the sperm abnormalities observed in the present study. However, further studies are required to confirm this hypothesis.

Some limitations of this study should be acknowledged. Hormonal analyses were limited to testosterone measurements, and additional endocrine markers, such as LH, FSH and prolactin, were not evaluated. Furthermore, although quantitative histological analyses were performed, stereological parameters, including the relative volume densities of germinal epithelium, tubular lumen and interstitium, were not assessed. Future studies incorporating comprehensive stereological approaches could provide a more detailed characterization of testicular structural alterations induced by trazodone. The 21‐day treatment period represents another limitation because it does not encompass the complete spermatogenic cycle in rats, which lasts approximately 54 days [19]. Therefore, the present findings should be interpreted primarily as evidence of early reproductive alterations following subacute trazodone exposure, and longer treatment periods encompassing a complete spermatogenic cycle will be necessary to determine the full extent and persistence of the effects on spermatogenesis. In addition, sperm motility was assessed by conventional microscopic analysis rather than by computer‐assisted sperm analysis (CASA), which would allow a more comprehensive evaluation of sperm kinetic parameters, including velocity and movement patterns. Finally, fertility assays and molecular analyses were not performed, limiting the mechanistic interpretation of the reproductive alterations induced by trazodone.

In conclusion, **t**razodone treatment induced adverse effects on male reproductive function in rats, characterized by testicular alterations potentially associated with oxidative stress. These effects were accompanied by impaired spermatogenesis and reduction in the quantity and quality of spermatozoa in the epididymis. Furthermore, trazodone impaired the contractility of the distal cauda epididymal duct, suggesting a potential interference with the ejaculatory process. To our knowledge, this study represents the first integrated evaluation of the effects of repeated trazodone administration on testicular morphology, sperm parameters and the contractile function of the distal cauda epididymal duct. By combining histological, functional and sperm analyses, our findings provide new insights into the potential mechanisms underlying trazodone‐induced adverse reproductive effects. Further studies are warranted to determine the translational relevance of these findings to humans and to assess their potential implications for male fertility.

## Funding

The Article Processing Charge for the publication of this research was funded by the Coordenação de Aperfeiçoamento de Pessoal de Nível Superior–Brasil (CAPES) (ROR Identifier: 00x0ma614).

## Ethics Statement

All experimental protocols were reviewed and approved by the Animal Ethics Committee (CEUA) of the Federal University of Rio Grande do Norte (Approval Certificate No. 352.031/2023; Protocol No. 031/2023) and carried out in compliance with the ARRIVE guidelines. In addition, the study was conducted in accordance with the Basic and Clinical Pharmacology and Toxicology policy for experimental and clinical studies [16].

## Conflicts of Interest

The authors declare no conflicts of interest.

## Data Availability

The data that support the findings of this study are available from the corresponding author upon reasonable request.
